# Older Worker Identity and Job Performance: The Moderator Role of Subjective Age and Self-Efficacy

**DOI:** 10.3390/ijerph15122731

**Published:** 2018-12-03

**Authors:** Francisco Rodríguez-Cifuentes, Jesús Farfán, Gabriela Topa

**Affiliations:** 1 Department of Medicine and Surgery, Psychology, Preventive Medicine and Public Health, Immunology and Medical Microbiology, Nursing and Stomatology, Rey Juan Carlos I University, 28300 Aranjuez, Madrid, Spain; francisco.rcifuentes@urjc.es; 2 Health Psychology Program, International School of Doctorate, National Distance Education University (UNED); 28040 Madrid, Spain; jfarfandiaz@gmail.com; 3 Department of Social and Organizational Psychology, National Distance Education University (UNED), 28040 Madrid, Spain

**Keywords:** group identification, older workers, job performance, psychological capital, self-efficacy

## Abstract

Older Worker Identity consists of the internalization of negative beliefs and attitudes towards aged employees by these same people. This research aims to explore the moderator role both of subjective age and self-efficacy in the relationship between older worker identity and job performance. The study was conducted with a panel design, including a sample of +40 Spanish workers (*n* = 200), with two waves (4-months interval). The findings supported the moderator role of subjective age in the relationship, while it failed to support the moderator role of self-efficacy. These findings underline that workers who actively manage their subjective age perceptions could age successfully at work. The implications of this study for counseling practices are discussed.

## 1. Introduction

At what age is one too old to work? Faced with this question, more than half of the workers reply that “it depends on the person”. Among the baby boomers, 68% of them give this answer. Moreover, if asked to respond with a specific age, most of them consider that at the age of 75, they would be too old to continue working [1]. This question seems to be in the center of the current debate about working longer, which is currently receiving the attention of the media and academia [2].

On the one hand, the interest in working longer is based on individual motivations. Many people over 55 expressed their desire to continue working beyond age 65 [1]. However, noticeably fewer workers manage to work at those ages [3]. On the other hand, from the social point of view, interest also is growing because of population aging and pressure on public pension systems, while the need for specialized manpower is pushing towards the extension of working life. The issue raises notable controversies because it involves at the same time older workers’ personal characteristics and organizations’ relevant outcomes, such as performance in the workplace [4].

At the center of this debate could be considered both the negative views of older workers, which they can internalize, and the employees’ task performance [5]. The present study has been developed under the overarching framework of the Model on the Interplay among age, social identity and identification at work, developed by Zacher et al. [6].

The workers’ own perceptions of age and self-efficacy can also play a role in this relationship. Thus, this study aims to analyze the relationship between identification with the group of older workers and employees’ task performance. In addition, we shall explore the moderator role of self-efficacy and subjective age in this relationship.

The findings of this study will allow us to establish whether older worker identity exerts any detrimental influence on task performance. In addition, they will allow us to establish whether it is possible to mitigate this influence through older workers’ subjective perceptions of age and the development of self-efficacy. People, businesses and governments need to have solid empirical evidence to design interventions that promote the extension of working life, both for individual well-being and to alleviate future economic difficulties.

### 1.1. Older Worker Identity and Task Performance

The negative stereotypes of older people nowadays seem widely spread [3]. Older workers often internalize this unfavorable view of themselves that prevails in their near environment. This is due to the fact that the experience of ageing at work takes place in a specific context in which the person behaves, and this affects self-perceptions. Daily interactions with co-workers and supervisors would transmit the repeated experience of unfavorable treatment, discrimination in career opportunities, and the lack of an offer of training for older workers in some cases [7].

Bearing in mind that, like all people, older workers identify with others based on a few shared traits, the concept of “older worker identity” (hereinafter, OWI) has been presented [8]. This is the term used to designate the extent to which a worker identifies with the older workers group and the consequent internalization of stereotypes and negative attitudes toward older workers by the older workers themselves. OWI is accompanied by the acceptance of negative characteristics of oneself, such as resistance to change, poor performance, or low work motivation [9].

OWI, therefore, includes two facets simultaneously. On the one hand, older people perceive that they are judged unfavorably and suffer discrimination from their coworkers and supervisors at work due to their advanced age [10]. On the other hand, these perceptions reinforce their view of themselves as old people, and, therefore, they assume as their own the negative aspects of older workers, such as slowness, inefficiency, low work motivation, lack of desire for training and promotion, and reduced performance [11]. The present conceptualization of OWI overlaps with the “age-related social identity” proposed by our theoretical model [6].

It is a proven fact that identification promotes the probability of acting consistently with the category with which the person is identified [12], so OWI can be an antecedent of undesirable behaviors, such as the decline in performance at work [13,14]. Previous studies have shown the existence of OWI [15] and its influence on the attitudes and behavior of older workers [16]. Specifically, research has found that OWI predicts the decline of job satisfaction, commitment, or performance [17,18,19]. In this sense, Snape and Redman [20] found positive and statistically significant relationships between OWI and intentions of early retirement, and other studies have proven its predictive power for absenteeism at work [21].

Regarding performance, it is currently common to consider it as a multidimensional construct [22], which four dimensions (task performance, contextual performance, adaptive performance, and counterproductive performance). Despite this fact, task performance seems to be the “core” of the concept. Task performance is the execution of the central tasks of the post, and it also seems to be the most stable dimension of total performance.

In relation to older workers, various studies support the hypothesis of reduced productivity [23]. However, this debate implies another one on the difficulty to define and measure work performance. As stated by Van Dalen et al. [24], depending on the criteria that are considered to measure workers’ outcomes, workers can be benefited or harmed. So, when referring to speed or intensity in monotonous or repetitive tasks, older workers may show a decline in performance or have more accidents and work-related diseases [25]. However, when referring to having experience, consolidated skills and social networks (which are developed over time), it can be seen that older workers achieve better performance than their younger coworkers [26]. Therefore, in this study, we want to explore the predictive power of OWI on task performance in workers over age 40. Hence, the first hypothesis of the present study is:

**Hypothesis** **1.** OWI will be negatively related to task performance.

### 1.2. The Moderator Role of Subjective Age and Self-Efficacy

The existing research to date has focused preferentially on the relation between older workers’ chronological age and performance but it does not show conclusive results, even stating that there is no relationship between age and performance [3]. Hence, among the different aspects that may have an impact on performance, the role of age does not seem so clear, despite its intuitive sense. Among other reasons, more recent research on performance has not incorporated a broader conceptualization of aging, but instead has focused almost exclusively on chronological age. Thus, previous studies in performance assessment did not investigate alternative age constructs, such as subjective age.

Subjective age refers to how young or old an individual perceives himself [27]. In accordance with Shore, Cleveland, and Goldberg [28], subjective age includes various components, such as the age people feel, their apparent age, the desired ideal age, and the age of the most similar people in terms of tastes, interests, and behaviors. Although many studies underline the relevance of considering chronological age as the most direct moderator in the relationships between organizational variables [29], some recent reviews dispute the role of chronological age in favor of subjective age [11].

In this sense, there is growing evidence of the role that subjective age can play in organizational performance [30] as well as in undesirable outcomes, such as absenteeism [31]. The moderator role of subjective age is tenable because it would be associated with the perception of having more specific experience for task performance and better adaptation to the work environment, thus resulting in a reduction of perceived work stress. On another hand, the evidence shows that subjective age may be linked to increased motivation, both for task performance [32] and for permanence in active work [33]. Therefore, in this research, we will explore the moderator role of age in the relationship between OWI and task performance, proposing the following hypothesis:

**Hypothesis** **2.** The relationship of OWI with task performance will be moderated by subjective age. As subjective age increases, the negative relationship between OWI and task performance would be stronger.

Despite the broad dispersion of negative stereotypes towards older workers, empirical research on their impact on performance is still incipient. As indicated by Chiesa et al. [5], the loss in productive terms seems to be mediated by the self-efficacy of the group of older workers, which offers an opportunity to mitigate reduced performance through an intervention to increase self-efficacy [34]. Self-efficacy consists of the personal beliefs in their own abilities to implement everything needed to perform a specific task in a satisfactory way [35,36]. The most recent theoretical models, close to positive psychology [37,38,39], include self-efficacy within psychological capital [40].

Researchers on self-efficacy show that people engage in the tasks about which they are confident that they have the necessary abilities to succeed. Thus, self-efficacy becomes a powerful determinant of behavior because it affects both the initial decision to perform it, the invested effort, the persistence shown, and even the final interpretation of the outcome. One of the key aspects of self-efficacy is its positive potential. Specific interventions to develop self-efficacy, or the broader intervention to increase psychological capital, have shown a beneficial impact for individuals, and also for organizations as a whole [41,42]. The benefits of self-efficacy have been reflected in results as varied as satisfaction and innovation [43], learning and creativity [44], turnover [45], health [46], quality of working life, and citizenship behaviors [47]. Finally, the following hypothesis is established:

**Hypothesis** **3.** The relationship of OWI with task performance will be moderated by self-efficacy. As self-efficacy increases, the negative relationship between OWI and task performance would be weaker.

**Figure 1:**
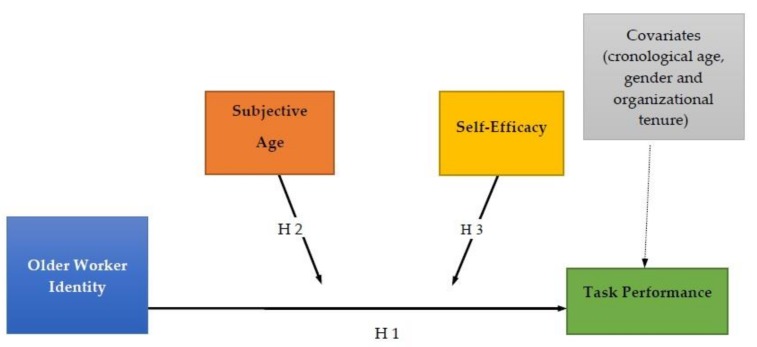
Model of proposed hypotheses.

The hypotheses are depicted in Figure 1.

## 2. Materials and Methods

### 2.1. Ethical Information

The Bio-Ethics Committee of the National Distance Education University approved the study protocol in accordance with the Declaration of Helsinki (protocol number 160504). In the present study, potential participants were informed of the objectives and the conditions of the study regarding voluntariness and anonymity, and the possibility of withdrawing from the research at any time without penalty. The only conditions to participate were being older than 40 years of age and having a status of employee. Those who finally decided to participate signed their consent and completed booklets containing the research questions.

### 2.2. Participants

The final sample of the study consists of workers in Spain aged over 40 years who answered the survey at two different times (hereinafter, Time 1 [T1] and Time 2 [T2]), with a 4-month interval. The interval was selected to reduce the potential threat of common variance bias. Thus, a total of 278 workers were surveyed at T1, and at T2, 200 completed questionnaires were collected (72% response rate). Of the sample, 56.5% were male, and the average chronological age was 48.11 years (SD = 6.93). Concerning educational level, 41% had university studies, 21% vocational training, 21% high school and 12% basic education and 4.5% were missing data. Regarding working status, 49.5% of the participants were employees, 29.0% middle managers, and 14.5% were managers or owners of the companies, while the rest were missing responses. The mean organizational tenure was 16.3 years (SD = 11.1). Most of the workers worked in companies with more than 200 employees (42.5%), 11% worked in companies of 50 to 200 employees, and the rest in companies of less than 50 employees. Concerning the sector, 18% worked in service companies, 11% in the energy sector, 10% in tourism, 7% in education or health, and the rest was distributed in various occupational areas.

### 2.3. Procedure

The study was disseminated among the potential participant companies through human resources consulting firms linked to the University with specific agreements to collaborate in research. The companies whose managers agreed to collaborate distributed the booklets with the surveys among their workers over age 40 at both times of data collection. Participants created a personal code especially for the study and handed in the completed questionnaires in a sealed envelope to the collaborators of the research team. Personal data were not known to the researchers.

### 2.4. Instruments

#### 2.4.1. Older Worker Identity

We used the OWI Scale [48]. It questionnaire has been adapted for Spanish population in a previous study [16], and its adequate psychometric properties have been proved. The ten items used request participants to self-rate their speed, their interest in professional development, and their flexibility at work. Examples of items are: “I think that I’m becoming slow to learn new tasks,” “I think that I am becoming less flexible and adaptable at work,” and “I think that I am not interested in updating and growing professionally.” The reliability of the instrument in previous studies [16] was adequate (0.80), and in the present study, it was 0.82.

#### 2.4.2. Subjective Age

We used the four-item scale of Shore, Cleveland, and Goldberg [28] asking people to indicate on a 5-point scale (1 = 16–25; 2 = 26–35; 3 = 36–45; 4 = 46–55; 5 = 56–75) the age that most closely corresponds to the way they feel (a), they look (b), the age of people whose interests and activities are most like theirs (c), and the age that they would prefer to be (d). Since there has not been a Spanish adaptation for this instrument, the research group translated it. The reliability of the instrument in this study was α = 0.70.

#### 2.4.3. Self-Efficacy

To evaluate this variable specifically referred to the tasks of the post, we used the Spanish adaptation [49] of the Self-efficacy subscale of the Psychological Capital Questionnaire (PsyCap), which measures four components of psychological capital [50]. This Spanish version of the PsyCap proven their adequate psychometric properties in the validation study [49]. The Self-efficacy subscale is made up of 3 items related to aspects of perceived confidence when undertaking a task (e.g., “I feel confident when I represent my work area at meetings with the directors”) and reached a value of Cronbach alpha of 0.75 in this study.

#### 2.4.4. Task Performance

We used the specific subscale of the Individual Work Performance (IWP) [51], which is made up of five items that represent the critical indicators identified by the authors for this dimension of performance: work quality, planning and organizing work, being result-oriented, prioritizing and working efficiently. Examples of items are: “I managed my work well so that it would be done on time,” “I have considered the results I should achieve in my work.” The reliability was α = 0.70 in this study.

For the measurements of OWI, task performance, and self-efficacy, the Likert-type response scale ranged from 1 (Completely disagree) to 5 (Completely agree).

### 2.5. Statistical Analyses

To test our hypotheses, we used the macro PROCESS for SPSS [52]. We applied Model 2, which estimates the relation of X (T1 OWI) on Y (T2 Task performance) with the moderation of the variables M (T1 Efficacy) and W (T1 Subjective Age) in the relation X→Y (T1 OWI → T2 performance). The hypothesis will be supported if, for different levels of the moderating variables, the effect of X on Y varies. The procedure was based on 5000 bootstrapping samples, with a 95% confidence interval. The procedure allows us to estimate the conditional effect of the independent variable on the dependent variable as a function of the effect of the moderators (mean and ±1 SD from the mean). When zero is not included in the 95% bias-corrected confidence interval, it may be concluded that the parameter is significantly different from zero at *p* < 0.05. Chronological age, gender, and organizational tenure were used as covariates.

## 3. Results

Before testing our model, a correlation analysis was conducted among the study variables. These results are reported in Table 1. Pearson’s correlations indicated that all significant relationships between the variables were in the expected direction. As expected, subjective age was highly correlated with chronological age and with the time the person has been in the company. In addition, OWI was significantly related to tenure in the company and gender. In this case, men had a greater tendency to identify with the characteristics of older workers than women.

### Moderation Analysis

The objective of this analysis is to test the hypotheses of this study. The model as a whole was significant, F(8, 192) = 9.41, *p* < 0.0000, R^2^ = 0.28. None of the covariates had significant effects on the prediction of task performance. The negative effect of older worker identity on task performance (B = −1.16, SE = 0.39, 95% CI (1.93, −0.38), *p* < 0.003) was significant, which supports Hypothesis 1 of the study.

**Figure 2:**
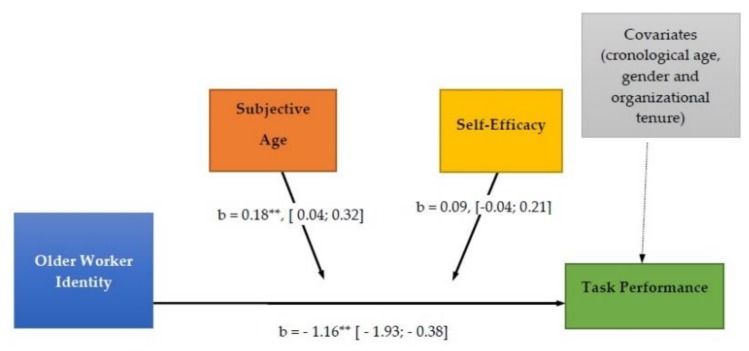
Results of moderation analysis. Note: [95% CI]; * *p* < 0.05; ** *p* < 0.01; *** *p* < 0.001.

Related to the moderating hypotheses, on the one hand, the interaction between subjective age and OWI was significant in the prediction of task performance, (B = 0.1849, SE = 0.07, 95% CI [0.04; 0.32], *p* < 0.0125), thus providing support for the second hypothesis, as Figure 2 shows.

On another hand, self-efficacy (B = 0.01, SE = 0.1509, 95% CI [−0.29, 0.319], *p* <.94) and the interaction between self-efficacy and OWI (B = 0.09, SE = 0.06, 95% CI [−0.04, 0.21], *p* < 0.16) were not significant in the prediction of task performance. These results do not support the third hypothesis of this study. The interaction of OWI with efficacy, despite not being statistically significant, improved the R^2^ of the global model, F(1, 191) = 1.92, *p* < 0.16, ∆R^2^ = 0.007, though its contribution was very small.

Both interaction terms improved the explanatory capacity of the model separately and concurrently. As for subjective age, its effect on task performance (B = −0.34, SE = 0.18) only showed a tendency of associated statistical significance [95% CI [−0.76, 0.01], *p* < 0.06]. The interaction OWI × Subjective Age was significant, F(1, 191) = 6.36, *p* < 0.01, ∆R^2^ = 0.02, and the contribution of the two interactions conjointly was also significant, F(2, 191) = 3.34, *p* < 0.03, ∆R^2^ = 0.03.

The conditional effect of OWI on task performance was significant at various levels of the moderating variables, but it lost its significance when subjective age and self-efficacy were high (both +1 SD; B = −0.075, SE = 0.0827, 95% CI [−0.2381, 0.0881], *p* < 0.3656). The largest effect was observed when subjective age and self-efficacy had lower levels, as shown in Table 2.

**Figure 3:**
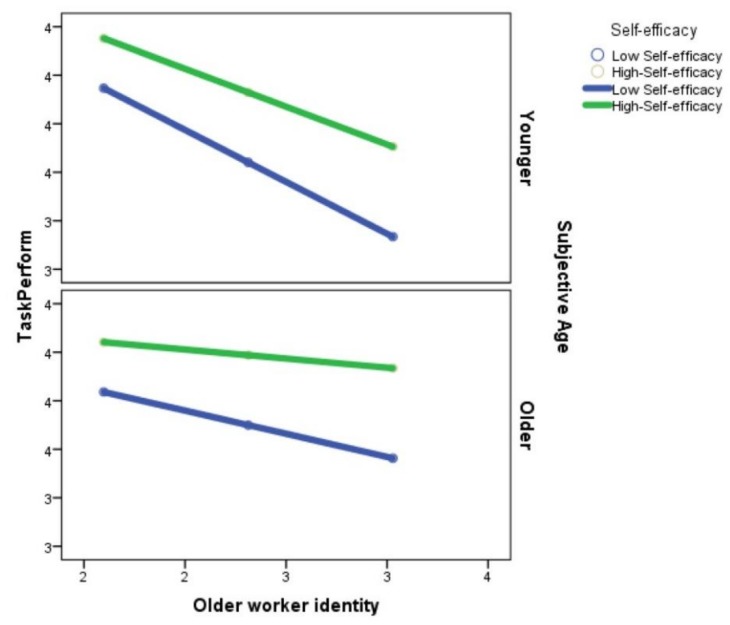
Plot diagram for the conditional effects of Older Worker Identity on task performance as a function of subjective age and self-efficacy.

These results indicate that the higher the self-efficacy, the better the task performance. However, this relationship was not the same at all levels of OWI or for all groups as a function of subjective age. The results can be seen in Figure 3.

This figure shows that the higher the OWI, the lower the performance. This negative relationship was found both groups, those who perceived themselves as younger and those who perceived themselves as older. But, for the former, the relationship was more intense, whereas OWI had less negative impact on performance when subjective age was higher. However, if self-efficacy was also high, the negative effect of OWI on task performance was also lower, thereby losing its statistical significance.

## 4. Discussion

Firstly, this work supports the hypothesis that the relationship between OWI and older workers’ task performance exists, and it is negative. However, our results are not limited to this finding, but instead provide data to understand that this relationship is moderated by other variables. On the one hand, OWI has a detrimental effect on performance in all cases, but the intensity of this influence varies depending on the subjective age and self-efficacy of the workers. In particular, subjective age acts as a moderator of that relationship. When workers perceive themselves as younger, but they internalize the negative traits of older people transmitted by the environment, their performance drops. This influence is verified more intensely for those who have low self-efficacy concerning the task. However, when workers perceive themselves as older, the negative effect of OWI on performance is lower. But even this result varies depending on self-efficacy, because for those workers who have stable beliefs about their abilities to perform the tasks, the negative impact of OWI on performance is even lower. These results may seem counter-intuitive, so they deserve a detailed discussion.

First, subjective age in the model is directly and negatively related to task performance. However, when analyzed together with identification with the group of older workers, the moderator effect can be seen. Workers may perceive their own OWI, involving the generalized idea of “reduction” of capabilities, but they may refuse to accept that this reduction affects them, and they may implement a series of responses to alleviate the potential deficit caused by age. In this sense, a large body of empirical evidence related to successful aging through selection, optimization, and compensation strategies seems to support that older workers can maintain adequate performance within organizations [53,54].

In relation to OWI, it can trigger an attributive process, for example, to disease, which serves to alleviate the negative effects on performance. Thus, stereotypes would not have the same impact for all groups [17] but would vary depending on the individuals’ behaviors developed to face with negative stereotyping. Further research can explore coping strategies, which may be focused on victimization, but also on the attempt to increase performance, as research on perceived discrimination in other areas seems to indicate [55].

On another hand, although the data do not support the hypothesis that proposed self-efficacy as a moderator in the relationship between OWI and task performance, when the conjoint model is observed, this influence can be seen. When analyzing the results, we see that the only interaction that is nonsignificant occurs when subjective age and levels of self-efficacy are high, as all the other interactions are significant, and the effect is greater as the values of both moderators decrease. In other words, the discrepancy between subjective age and OWI exacerbates the negative impact on performance and, although high self-efficacy is insufficient to explain the improvement in performance in the model, its lack seems to worsen performance [56].

In summary, this study highlights the importance of individuals’ perceptions. On the one hand, as shown in Figure 3, OWI has crucial importance: If workers do not identify with the group of older workers, their performance reaches higher levels [57]. On the other hand, we note the importance of aspects like subjective age, because when people maintained the characteristics of older workers’ stereotypes and they could account for them in their own self-perception as older people, their work performance was somewhat protected from reduction.

### 4.1. Limitations of the Study

This study has several limitations that must be acknowledged. First, it is worth noting that the measure of performance used in this study only refers to task performance. Other measures of performance may be affected in the opposite direction and thus, would alleviate the negative effects of OWI. In addition, in some specific posts, being older may be advantageous for task performance, or a nonlinear relationship or an inverted U-shaped function might be verified.

As in the present study only have been included measures of age-related social identity, following the distinction recently proposed by Zacher and colleagues [11], we could not provide empirical support for the assertion regarding the moderation of age meta stereotypes in the relationship between age related social identity and performance.

Secondly, regarding the lack of significance of self-efficacy, this might be caused by the characteristics of the study sample. The self-efficacy scale items are formulated in terms of confidence when discussing or representing work areas, but nearly half of the respondents are employees, so these skills may not be essential when measuring their task performance.

The third limitation refers to the characteristics of the sample, as the selection of the participants in this study was not random, but instead, we used convenience sampling, and this may have biased the results. Fourth, and although in the analyzed literature, the results of research in different countries are usually consistent, our findings may not be transferable to other cultural environments due to the existence cultural differences. The extra effort to achieve higher performance may be rated negatively in certain cultures, as well as the desire to maintain a high performance to continue working beyond the age of retirement. In fifth place, as we only used self-report measures, despite the confidentiality of responses, there is always the threat of social desirability bias.

### 4.2. Suggestions for the Extension of Working Life

The present study provides evidence about the importance of how workers within the company feel, represented herein by the role of OWI, beyond the mere objective conditions of the post. In this sense, if organizations want to prolong the working life of their employees, they should pay special attention to their workers’ appraisals, especially those of the older workers, because a climate for successful aging favors the individual application of strategies to alleviate the negative effects associated with age [58]. Interventions in companies to teach their workers alternative ways of dealing with new problems that arise in their jobs can improve and reduce the negative impact of aging on organizational outcomes. Thus, a cognitive intervention that highlights the positive aspects of older people versus the negative aspects and that enhances generational diversity can have a positive impact on outcomes. Promotion of organizational identification that unites all the members of the company, regardless of age, seems a simple and effective means to ensure the survival of the company and the extension of the working life of its members.

As noted in other works [5,39], interventions to counter the impact of negative stereotypes can focus on increasing self-efficacy. Thus, in line with Salanova et al. [59], self-efficacy is related to positive spirals that translate into improvements both of commitment to the company and positive motivation, perhaps through organizational metacognitions [60]. But this study also poses a new way because interventions could focus on subjective age to offset the decline in performance. Although some lines of research are currently questioning the usefulness of the construct of subjective age [56], the evidence supporting the influence of this variable seems solid and continues to grow [61].

At the same time, as an anonymous reviewer suggested, other related topics that can affect our findings should be considered. First, different kinds of occupations are associated with specific occupational risks. In this sense, relevant levels of physical job demands can affect older workers inducing health problems that reduce task performance and worsen self-perceptions of age. As our study included participants with different organizational levels, the OWI could be influenced by their specific roles as employees or owners, for instance. Secondly, employees’ personality traits and attitudes or behaviors can influence their job demands perceptions and performance, as other studies suggested [62]. Thirdly, while it has been mainly considered that stress and job demands could negatively affect employees’ health status, more recently it has also been suggested that work can positively affect workers’ well-being by improving cognitive functioning and perceived health [63].

This work shows that it is possible to encourage the extension of working life and have a positive impact on the company’s outcomes through the workers’ personal resources, as social support, religious endorsement or career commitment [64]. In this sense, fostering the improvement of aspects like subjective age through training in observation of the positive features can lead to older workers’ continued engagement, with the consequent advantage of having access to their experience in the formation of new staff members [65].

## 5. Conclusions

The study provides further evidence of the negative relationship between older worker identity and task performance. In addition, it examines the relationship between self-efficacy and subjective age, with subjective age being a moderator of the relationship between self-efficacy and task performance, producing a buffer effect on the reduction of performance when the person subjectively perceives him/herself as a member of the group of older workers.

## Figures and Tables

**Table 1:** Descriptive statistics and correlation matrix. (*n* = 200).

Variables	M	SD	1	2	3	4	5	6	7
1. Gender	1.41	0.52	n.a						
2. Chronologic Age	48.11	6.93	−0.09	n.a					
3. Organizational tenure	16.25	11.13	−0.12	0.53 **	n.a				
4. Older Worker Identity	2.31	0.72	−0.16 *	0.10	0.15 *	0.82			
5. Self-efficacy	3.94	0.67	0.02	−0.06	0.06	−0.16 *	0.75		
6. Subjective Age	3.02	0.66	−0.08	0.67 **	0.32 **	0.11	−0.13	0.70	
7. Task performance	3.82	0.48	0.07	−0.5	−0.3	−0.42 **	0.34 **	−0.1	0.70

Note: Gender (1 = Male); Values in the diagonal are reliabilities of the variables. * *p* < 0.05; ** *p* < 0.01; *** *p* < 0.001. n.a.: Not available.

**Table 2:** Conditional effect of OWI on Task Performance at values of Subjective Age and Self-Efficacy.

Subjective Age	Self-Efficacy	Effect of OWI on Task Performance	SE	*t*	*p*	LLCI	ULCI
2.3694	3.2638	−0.4334	0.0890	−4.8673	0.0000	−0.6091	−0.2578
2.3694	3.9367	−0.3736	0.0638	−5.8561	0.0000	−0.4994	−0.2478
2.3694	4.6095	−0.3137	0.0620	−5.0635	0.0000	−0.4360	−0.1915
3.0250	3.2638	−0.3122	0.0662	−4.7162	0.0000	−0.4427	−0.1816
3.0250	3.9367	−0.2523	0.0438	−5.7612	0.0000	−0.3387	−0.1659
3.0250	4.6095	−0.1925	0.0555	−3.4660	0.0007	−0.3021	−0.0830
3.6806	3.2638	−0.1909	0.0739	−2.5853	0.0105	−0.3366	−0.0453
3.6806	3.9367	−0.1311	0.0662	−1.9791	0.0492	−0.2618	−0.0004
3.6806	4.6095	−0.0713	0.0834	−0.8548	0.3938	−0.2357	0.0932

Note: Values for both moderators are the mean and plus/minus one SD from mean.
